# Discovery of Several New Families of Saturable Absorbers for Ultrashort Pulsed Laser Systems

**DOI:** 10.1038/s41598-019-56460-5

**Published:** 2019-12-27

**Authors:** Syed Asad Hussain

**Affiliations:** Department of Engineering Science, University of Oxford, Parks Road, Oxford, OX1 3PJ UK

**Keywords:** Fibre lasers, Mode-locked lasers, Ultrafast lasers

## Abstract

Saturable Absorber (SA) is a key element of any passive mode-locked laser system to provide ultrashort laser system. So far various materials have been proposed that could be used for this purpose. However, the field is still looking for new ways to make the fabrication process easier and cost-effective. Another challenge in testing mode-locked laser systems using various SA samples is the lack of knowledge in preparing these by laser physicists given this is outside their remit of expertise. In this study, we have proposed a novel method to produce these SAs from plastic materials and glycol. Our new method relies upon increase in thickness up to a value where the modulation depth is enough to give stable ultrashort pulses. Although we have shown this method for four materials; similar approach could be applied to any material. This will open the door of unlimited families of SAs that could be easily prepared and applied without any prior knowledge in material sciences.

## Introduction

Lasers are now frequently used in various commercial and scientific instruments notably surgery, spectroscopy, telecommunication, microscopy to name few^1–6^. Since its discovery, it has seen various extensive development in its various properties such as size, power, wavelength and so on^7^. One of these properties is to get mode-locked ultrashort pulses that helps to get high peak powers that are useful for applications like material processing, microscopy and laser surgery^8,9^. One mechanism to achieve mode-locking is through passive technique based on SA.

For any material to qualify for SA:

1. The material should have normal absorption that will give rise to non-linear absorption at the laser wavelength. According to Beer lambert law the linear absorption increases as we make the sample thicker. Whereas for a constant sample thickness, non-linear absorption decreases as we increase the power. This is due to the fact that in this case we are providing more photons to the sample that has finite number of electrons^10^. When the number of photons become significantly higher than the electrons in the SA, the sample becomes transparent for high intensity laser pulse and opaque for low intensity noise pedestal/side lobes of the laser pulse. The difference between minimum and maximum non-linear transmission is commonly known as contrast or modulation depth (ΔR)^10^. For fibre lasers working in negative group velocity dispersion, the minimum value of modulation depth was found to be around 2%^11^. In comparison to this, there is no maximum limit on modulation depth. However, Hönninger *et al*. provides us a good guideline on this^12^. According to their guidelines, the product of saturation energy of laser gain material (E_L_) and absorption (E_A_), and modulation depth (ΔR) should be lower than energy inside the cavity (E_p_), i.e. E_p_ > E_L_E_A_ΔR. By satisfying this criteria, the laser will give mode locked pulses, otherwise Q-switched pulses will be achieved, please see Fig. 8 in ref. ^12^. Increasing the modulating depth beyond the Q-switched regime, the laser will only provide continuous wave laser and by increasing it further the laser will not work.
2. The electrons after excitation to conduction band must return to the valance band within the cavity roundtrip time so that the returning pulse could again encounter same or significant amount of non-linear absorption^10^. In the case when the electrons are not completely returning to the valance band, we should consult ref. ^11^ that has values of modulation depth a laser should encounter in each roundtrip to provide a stable laser operation. Failing to achieve this, the laser cavity would not provide modulation of signal that is important to lock cavity’s longitudinal modes that results in an ultrashort pulse.
3. Another important characteristic of a SA is the damage threshold. The SA must withstand high peak powers of the laser cavity.
4. Finally, it must be easy to integrate inside the laser. The SA should be made in such a way that it goes easily with the design of the laser cavity. In the case of solid-state lasers, Vertical-External-Cavity Surface-Emitting Laser (VECSEL) and mode-locked integrated external-cavity surface emitting laser (MIXSEL) semiconductor, SA is normally integrated into a mirror that has several layers of dielectric materials to obtain desired electric field on the SA^10,13^. For other materials several groups have used drop casting or spin coating^14,15^. In fibre lasers, SA is normally mixed with a host material, dried at room temperature and a small piece is sandwiched between two fibre tips^16^. Other famous techniques in fibre technology is deposition on side polished fibre, back coupling by a SA mirror and drop casting on a micro fibre^16^. All these techniques have proved to be capable of integrating the SA inside the cavity.

A lot of materials have been used for this purpose. In this landscape semiconductors were extensively studied in the past^10^. Followed by it, there are materials such as graphene^17^, carbon nanotubes^18^ and other two-dimensional materials which have shown similar behaviour^19^. This results in several reports in terms of publications, thesis and patents costing several million euros in research funding. Now, various other materials such as topological insulators^20^, titanium dioxide^21^ and two-dimensional transition metal carbides and nitrides (MXenes)^22^ to name a few, are being used as other possible alternatives. Preparing these might not be possible for some research teams who have limited knowledge of material sciences. Moreover, except for graphene that could be produced easily by scotch tape, other materials are complicated to make and their fabrication requires training and experience. Recently relatively simple and inexpensive technique incorporating alcohol has been used for this purpose^23^. Still it is liquid at ambient temperature and is not suitable for humid and remote applications. In this report, we have shown that mode-locking could be achieved with polymeric materials such as Poly Styrene Methyl Methacrylate (SMMA), Polyvinyl Alcohol (PVA), Sodium Salt of Carboxymethylcellulose (CMC) and glycol alone.

We can achieve this by increasing the thickness of these materials until the absorption is enough to give mode-locked pulses satisfying condition 1 above. Although various properties could be tested in other specialized setups (i.e. damage threshold^24^ and relaxation time by pump-probe^25^), our study is focused on condition 1 as the material will not provide mode-locked pulses if other conditions are not fulfilled. This approach is cheap, easy to make, where no special training or prior knowledge in material science is required, and same method could be applied to any material around us, giving us with an infinite number of possible SAs that could be used, provided they have all the other properties mentioned earlier. This technique is not only limited to these plastic materials but could be extended to materials that are liquids at room temperature. In this report, we have used Thulium doped laser working at 1.87 μm laser and obtained mode-locked pulses by using SMMA, PVA, CMC and glycol, but these materials could be applied to other laser gain materials as well.

## Results and Discussion

**Figure 1:**
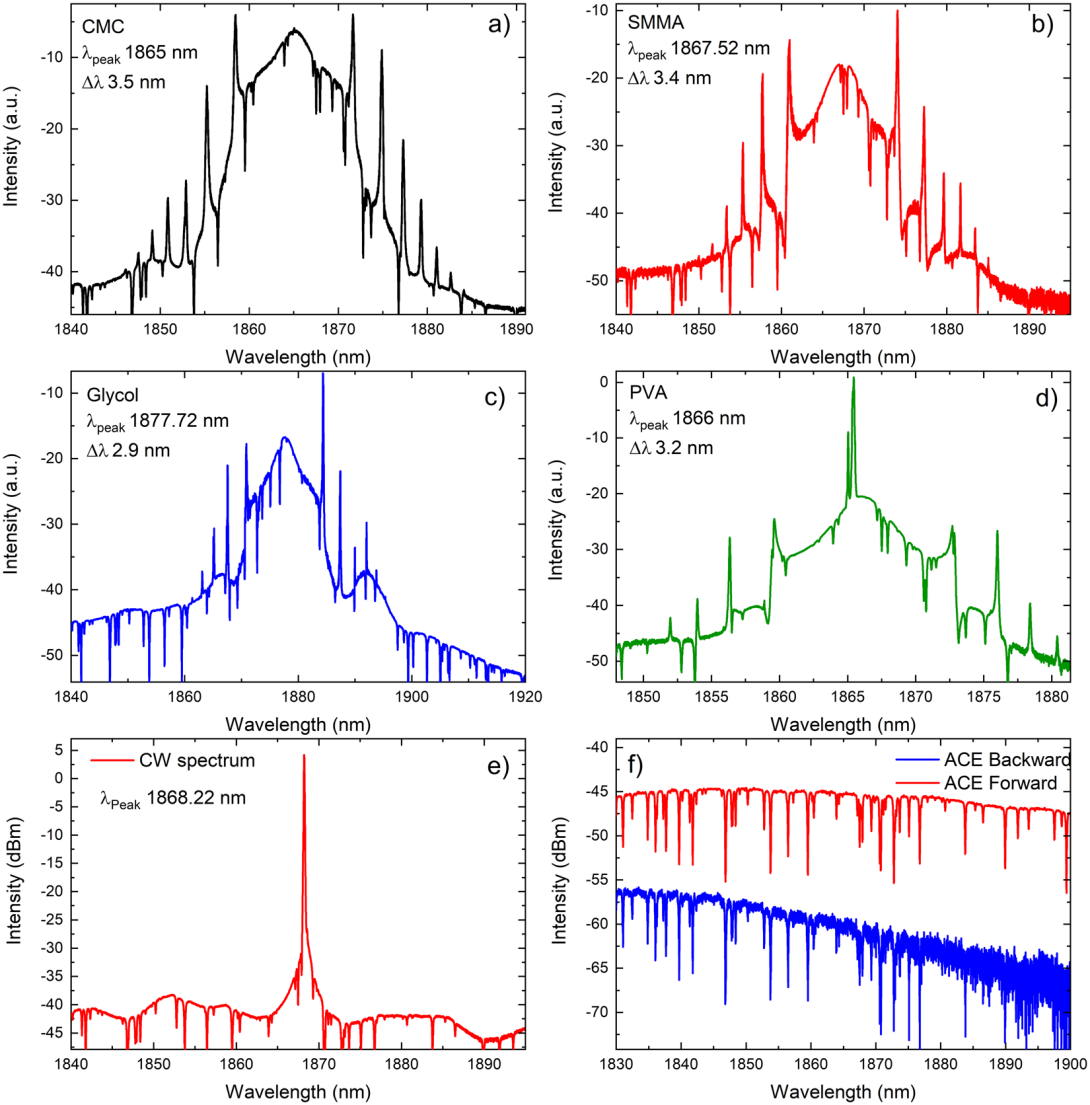
Optical spectrums obtained for various conditions. Mode-locked spectrum obtained by using (**a**) CMC, (**b**) SMMA, (**c**) Glycol and (**d**) PVA. CW spectrum obtained without any SA, Fig. 5(e). Forward and Backward ACE spectrums, (**f**).

The samples were tested systematically one by one into the cavity. The threshold for mode-locking for each sample is presented in Table 1. After mode-locking, the laser provided mode-locked pulses in the 1.8 μm region. Figure 1 shows the obtained spectrum for each case. To make a comparison without the SA (Fig. 1e), we obtained the spectrum at 1.2 Watt. In this case, we obtained continuous wave (CW) spectrum. The dips in the spectrums are due to atmospheric absorption. This is confirmed by obtained forward and backward amplified spontaneous emission (ASE) of the cavity and contain same absorption signature (Fig. 1f). The amplitude of backward ASE was lower than forward as discussed in other reports^26^. All the presented spectrums were obtained by a commercial spectrometer (Yokogawa, Japan). Figure 1 also presents width of each spectrum which is around 3 nm. The presence of Kelly side bands in the laser spectrum confirms the laser was working in solitonic regime^27^.

**Table 1:** Different parameters observed during the study.

	CMC	SMMA	Glycol	PVA
Threshold for mode-locking (Watt)	1.15	0.95	0.83	0.85
λ_peak_ (nm)	1865	1867.52	1877.72	1866
Δλ (nm)	3.5	3.4	2.9	3.2
ACF (ps)	0.96	1.66	1.18	1.63
Pulse duration (ps)	0.48	1.08	0.59	1.06
Fit type	Lorentz	sech^2^	Lorentz	sech^2^
TBP	0.14	0.32	0.15	0.29
Pulse train separation (ns)	64	64	64	64
Repetition rate (MHz)	15.6	15.6	15.6	15.6

**Figure 2:**
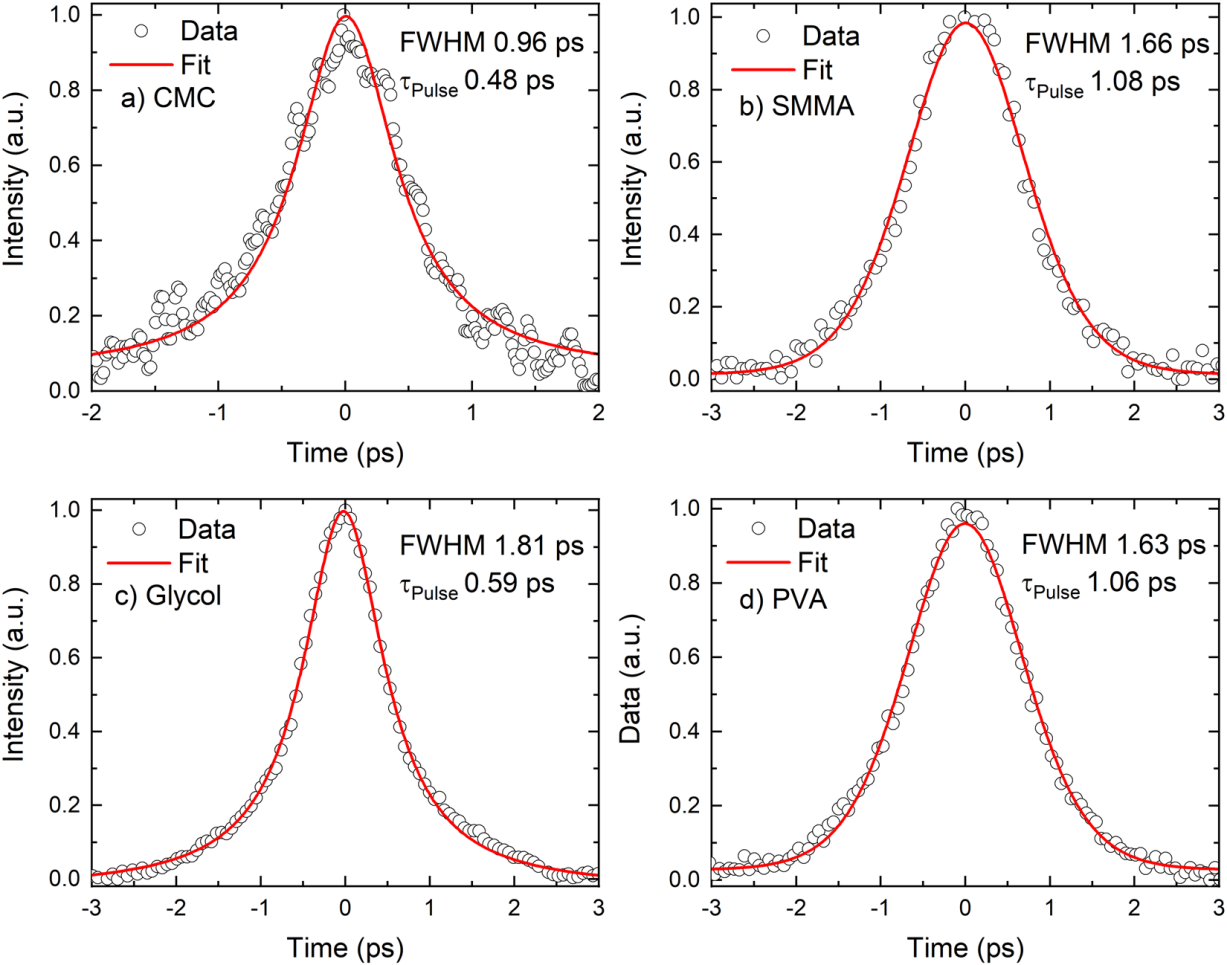
Autocorrelation traces obtained from the mode-locked laser (**a**) CMC, (**b**) SMMA, (c) Glycol and (**d**) PVA.

To obtain the time-bandwidth product (TBP), we have obtained pulse duration by using a commercial autocorrelator (APE, Germany). The obtained autocorrelation traces are presented in Fig. 2. Depending upon the pulse profiles we have used Lorentz profile (CMC and PVA) and sech^2^ profile (SMMA and Glycol). All pulses were close to transform limited values^9^ except for PVA which we think could be due to slight asymmetry of the pulse.

**Figure 3:**
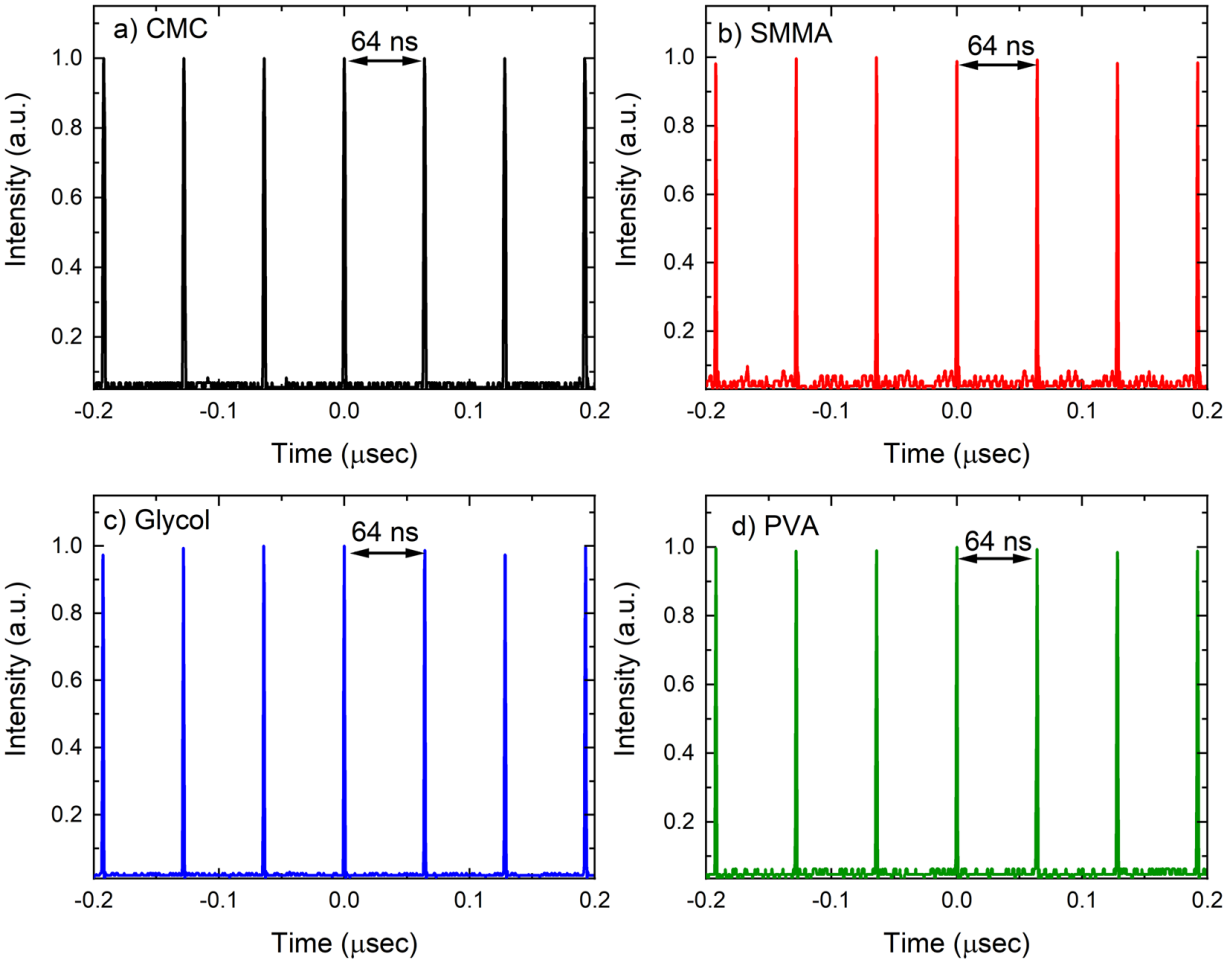
Pulse train obtained in various cases (**a**) CMC, (**b**) SMMA, (**c**) Glycol and (**d**) PVA. In all situations we have obtained a pulse separation of approximately 64 ns.

**Figure 4:**
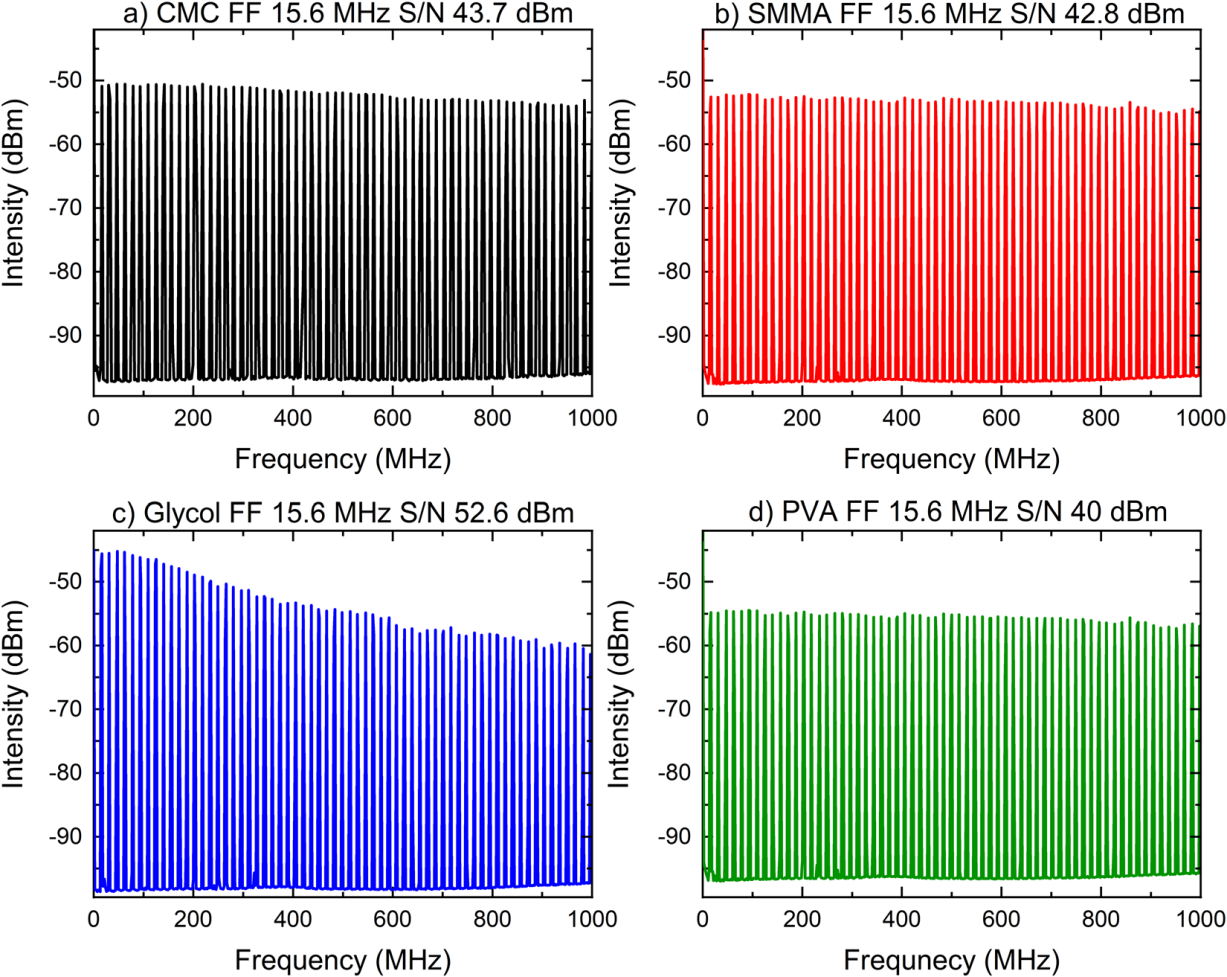
Radio frequency spectrums of the laser with (**a**) CMC, (**b**) SMMA, (**c**) Glycol and (**d**) PVA. The Fundamental Frequency (FF) was 15.6 MHz. Moreover, signal to noise ratio (S/N) were above 40 dBm.

Pulse trains were obtained by using a fast photodetector (Newport, United States), Fig. 3. The obtained signal was observed by an oscilloscope (Keysight, United States). The same signal was also investigated for radio frequency spectrum (Anritsli, Japan) with a span of 1000 MHz. In all cases we have obtained a repetition rate of 15.6 MHz (Fig. 4) and pulse separation of 64 ns, Table 1.

## Conclusion

In conclusion, in this paper, we have presented a method to obtain inexpensive materials to be used as SAs inside a laser to provide mode-locked ultrashort pulses. Provided the material has absorption at the laser wavelength, the thickness of the material should increase until its modulation depth is as per the requirement of the laser. This methodology could be applied to any substance to be used as a SA. In our paper we have selected plastic materials and glycol as they are inexpensive and easy to obtain. Another feature of the presented study is that preparation of the SA samples could easily be adopted by laser scientists who are not particularly informed about the skills required in material science. Therefore, they do not need to rely on external collaborations which saves time, cost and efforts. A comparison with other mode-locking methods (active and passive), SAs (semiconductor, two-dimensional materials, carbon nanotube and so on), and various laser techniques (solid-state, waveguide, VECSEL and MIXSEL) are not presented as they are beyond the scope of this paper^28–38^. This paper focuses on SAs being used in a fibre laser, which has the merit of being compact and portable. The samples that we used in this study were able to produce pulse duration in ultrashort time scale and were able to provide tenth of mega pulses out from the laser cavity at the wavelength around 1.87 μm. We believe that the presented study will provide new families of SAs by using the methodology that helps the researchers on not relying on expensive and sometime toxic materials that has already been presented in the literature as a SA.

## Materials and Methods

### Preparation of SAs

SMMA freestanding film is produced by dissolving SMMA in (N-Methyl-2-Pyrrolidone) NMP with a concentration of ~0.5 wt%. The SMMA solution in NMP is casted on a glass substrate and is vacuum dried at 80 °C overnight. Freestanding SMMA film of thickness ~92 μm is produced. On the other hand, PVA and CMC are dissolved in water with a concentration of ~1% each and are oven dried at 80 °C and the solution was casted on glass to produce films of thicknesses ~98 and ~186 μm respectively. Film obtained in this way is easy to integrate like other methods discussed in point 4 in the introduction. All the chemicals were obtained from Sigma-Aldrich and have purity >99%.

**Figure 5:**
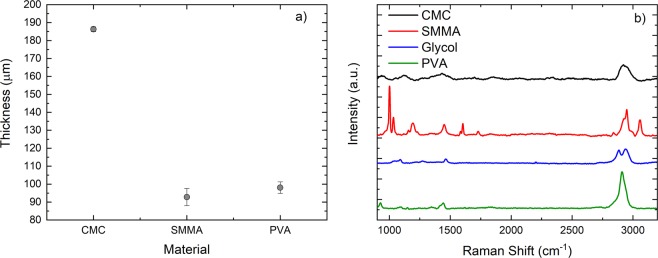
(**a**) Thickness of samples used in the study. (**b**) Raman spectrums of all the samples used in mode-locked laser.

**Figure 6:**
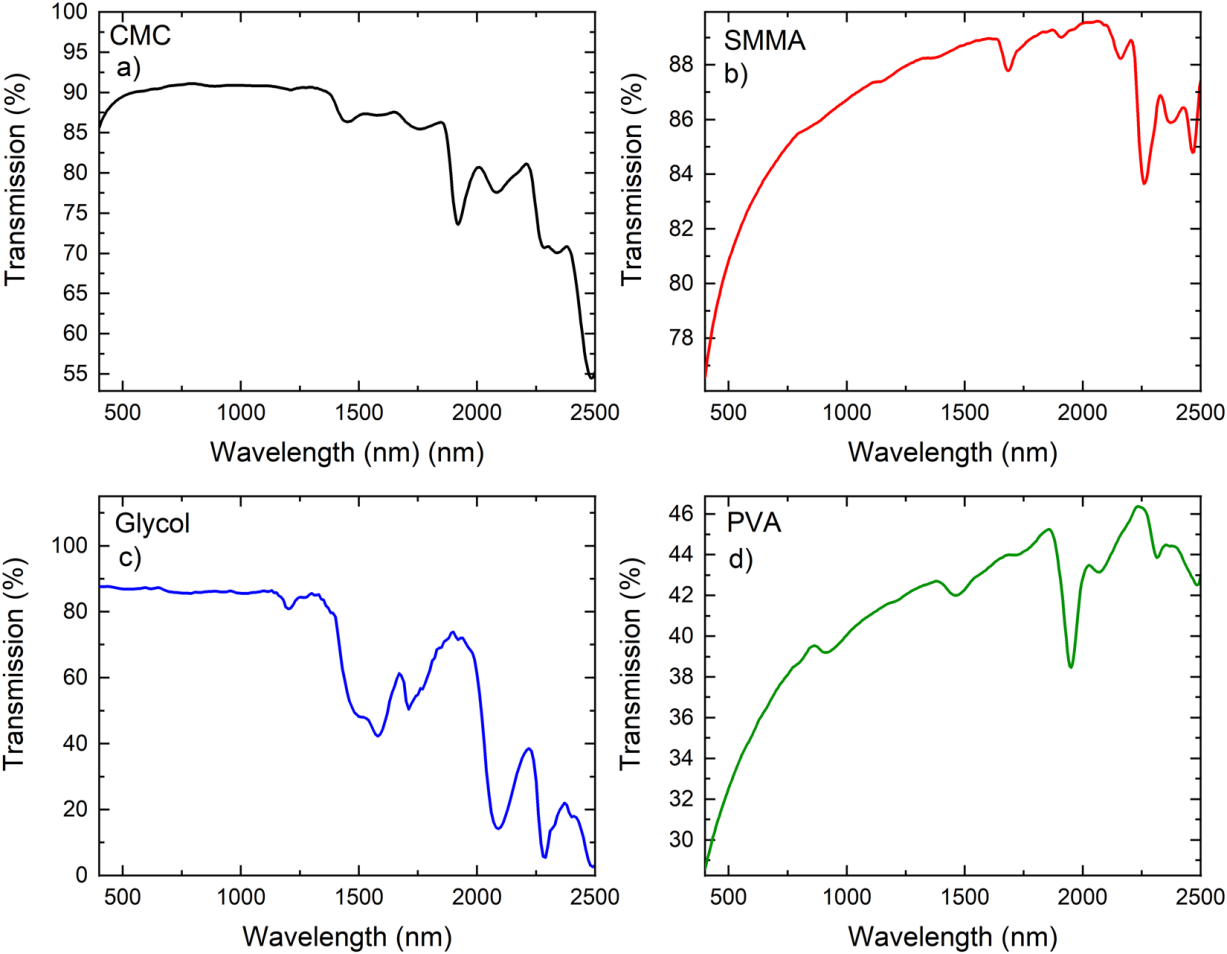
Transmissions of the samples used in the study. (**a**) CMC, (**b**) SMMA, (**c**) Glycol and (**d**) PVA.

Different thicknesses were used in the cavity until the cavity was mode-locked. To control the thickness we have used various areas and volumes to dry the polymers. Figure 5(a) shows thicknesses of various materials used in the study. The mean thickness of 125 μm was enough to give mode-locking in our laser system. It was not possible to measure the thickness of Glycol sample. We believe that it was around the same value. The samples were then tested by using Raman spectroscopy and obtained results are presented in Fig. 5(b). The Raman spectrums were obtained by using a commercial Raman spectrometer (Horiba, Japan). For the study we have selected the range from 110 to 3200 cm^−1^ by using 514.5 nm excitation and using a 100X microscope objective (Olympus, Japan). All the Raman spectrum agree well with the spectrum reported earlier^39–41^. By using a spectrometer (Agilent, United States) we obtained the transmissions of CMC, PVA, Glycol and SMMA. The obtained results are presented in Fig. 6. All samples have absorption at around 1.87 μm, that is the wavelength at which the SAs were giving mode-locked laser pulses. All samples tested here for transmission were used in the laser cavity except Glycol. The transmission of Glycol presented here is to give the reader a flavour about the typical transmission of this sample, Fig. 6(c). The samples also show various peaks at other wavelengths notably at 1.5 and 2 μm. At these wavelengths we have Erbium and Holonium gain materials^42,43^. This suggests that these samples could also be used as a SA for these laser gain materials. Unfortunately, the samples do not have significant absorption peaks at 1000 nm at which other laser gain materials exists.

### Non-linear transmission

**Figure 7:**
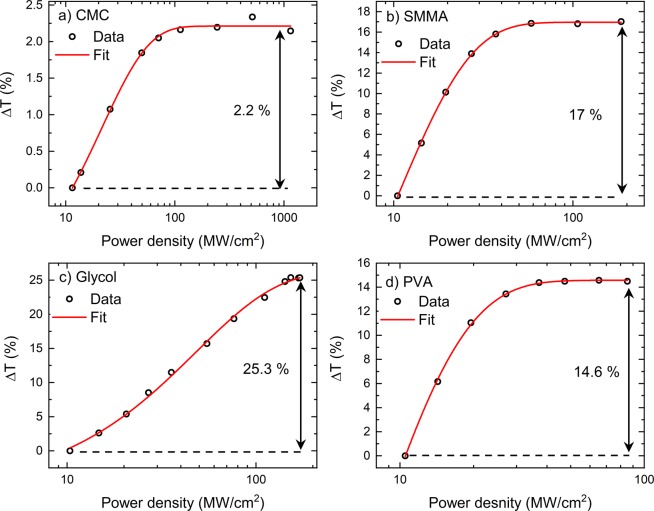
Non-linear transmissions of the samples used (**a**) CMC, (**b**) SMMA, (**c**) Glycol and (**d**) PVA. For the samples we have obtained modulation depths from 2.2 to 25.3%. These were obtained by using a commercial mode-locked laser working at 1865 nm providing 200 fs long pulses at a repetition rate of 80 MHz.

To obtain non-linear optical transmission, we used commercial Optical Parametric Oscillator (OPO) system (Coherent, United States). This system was pumped by a Ti:Sapphire laser with 200 fs long pulses at a repetition rate of 80 MHz. The output of the laser was coupled into a fibre that had an FC/PC end. A small piece of SA bigger than the core of the fibre (8 μm) was sandwiched with another FC/PC fibre by using a fibre connector. In these experiments the SAs were examined at 1865 nm. Figure 7(a–d) shows the obtained results. For the samples tested, we have achieved modulation depths from 2.2 to up to 25.3%. The values of these modulation depths are enough to provide ultrashort pulses from fibre lasers that have high gain in comparison to solid state lasers^44,45^. Since glycol is liquid at room temperature and the required thickness of liquid for mode-locking was unknown. To overcome this problem, one of the FP/PC fibres was attached with the fibre connector and connector was filled with glycol. The other connector was slowly moved towards the first connector, and this movement was seized as soon as we obtained mode-locked operation of the laser cavity. Without delay the fibre FC/PC, connector and fibre FC/PC assembly was disconnected from the laser cavity and used for non-linear transmission, Fig. 7(c). Followed by this, we fully inserted the fibre into the connector and no mode-locking operation was observed.

### Ultrashort fibre laser at 1.87 μm

**Figure 8:**
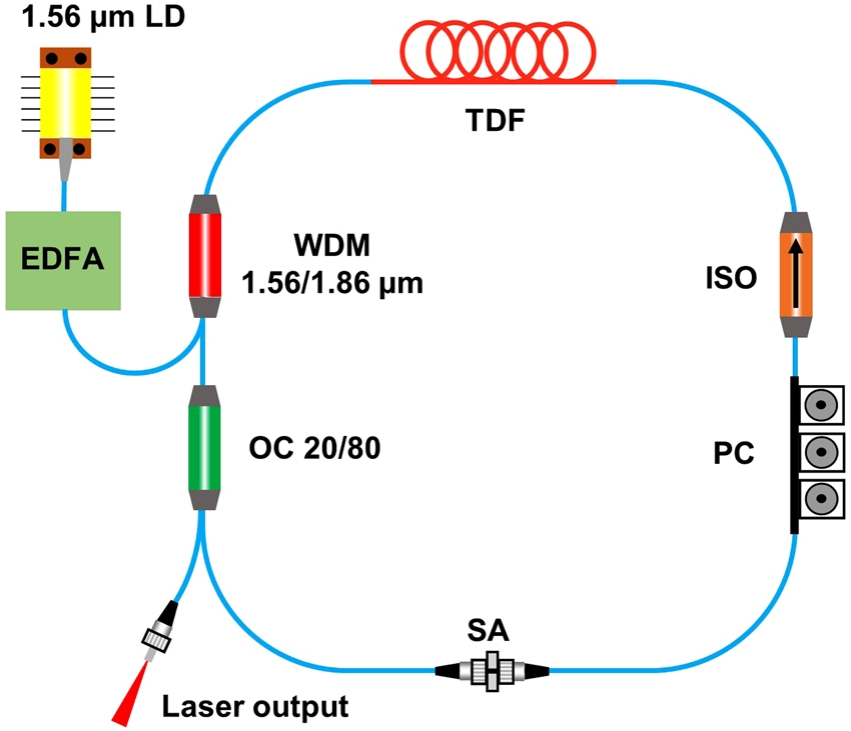
Schematic of the cavity used in the experiment. LD: Laser diode; EDFA: Erbium doped fibre amplifier; WDM: Wavelength division multiplexer; TDF: Thulium doped fibre; ISO: Isolator; PC: Polarization controller; SA: Saturable absorber OC: Optical coupler. Twenty percent of the power was taken out from the cavity for the characterisation of the laser (Image created with MS PowerPoint).

The 1.87 μm cavity was made by using a ~3 m long Thulium (Tm) doped fibre, Fig. 8. This was pumped by an amplified erbium doped fibre amplifier (EDFA) at 1.56 μm connected to the cavity by using a wavelength division multiplexer (WDM). To ensure a unidirectional flow, fibre isolator was incorporated to ensure a unidirectional circulation of laser pulses whereas polarising controllers (PC) helped to control the polarising state and provide stability to the laser pulses. The output power was taken out by using a 20% port of the output coupler. SAs were systematically sandwiched between two FC/PC connector ends. These fibre ends were connected by using a fibre connector^46^.

## Data Availability

Data used in this study can be provided from the author upon receiving a suitable request.
